# Appendix Q: Recommendations for Developing Molecular Assays for Microbial Pathogen Detection Using Modern *In Silico* Approaches

**DOI:** 10.1093/jaoacint/qsaa045

**Published:** 2020-07-30

**Authors:** John SantaLucia, Shanmuga Sozhamannan, Jason D Gans, Jeffrey W Koehler, Ricky Soong, Nancy J Lin, Gary Xie, Victoria Olson, Kristian Roth, Linda Beck

**Affiliations:** 1 Wayne State University and DNA Software, Inc; 2 Logistics Management Institute supporting Defense Biological Product Assurance Office (DBPAO), Joint Project Leads (JPL) Chemical, Biological, Radiological, and Nuclear Defense (CBRND) Enabling Biotechnologies (EB); 3 Los Alamos National Laboratory (LANL); 4 U.S. Army Medical Research Institute of Infectious Diseases (USAMRIID); 5 U.S. Food And Drug Administration (FDA); 6 National Institute of Standards and Technology (NIST); 7 U.S. Centers for Disease Control and Prevention (CDC); 8 Joint Research and Development, Inc. (JRAD) supporting Joint Program Executive Office (JPEO) JPL CBRND EB; Deputy Under Secretary of the Army, Test and Evaluation (DUSA TE)

## Abstract

We describe the use of *in silico* approaches to improve the process of molecular assay development and reduce time and cost by utilizing available databases of whole genome pathogen sequences combined with modern bioinformatics and physical modeling tools. Well-characterized assays are needed for accurately detecting pathogens in environmental and patient samples and also for evaluation of the efficacy of a medical countermeasure that may be administered to patients. The polymerase chain reaction (PCR) remains the gold standard for pathogen detection due to the simplicity of its instrumentation, low cost of reagents, and outstanding limit of detection (LOD), sensitivity, and specificity. However, creation of such PCR assays often involves iterations of design, preliminary testing, and thorough validation with clinical isolates and testing in relevant matrices, which can be time consuming, costly, and result in suboptimal assays. Since formal validation (e.g., for Emergency Use Authorization [EUA] or Food and Drug Administration [FDA] licensure) of an infectious disease assay can be very expensive and can require extensive time of development, having a well-designed assay up front is a critical first step. Yet, many assays described in the literature utilized limited design capabilities and many initially promising assays fail the validation process, resulting in increased costs and timelines for successful product development. While the computational approaches outlined in this document by no means obviate the need for wet lab testing, they can reduce the amount of effort wasted on empirical optimization and iterative redesigns and also guide validation studies. The proposed computational approaches also result in higher performing assays with better sensitivity, specificity, and lower LOD and reduce the possibility of assay failure due to signature erosion. To provide clarity, an extensive glossary of defined terms is provided.

## 1 Background and Rationale

Nucleic acid-based assays, such as real-time PCR, are the mainstay of clinical diagnostics and biosurveillance. A typical PCR assay design begins with computational (“*in silico*”) identification of a unique region (signature) that can support the binding of primer and probe sequences for target-specific amplification as a means of detecting the presence of the target organism. This step is followed by wet lab testing of the primers and probes using genomic deoxyribonucleic acid (DNA) or reverse transcribed ribonucleic acid (RNA) and performance-optimization of selected assays. In addition, extensive testing of the assay in the intended clinical matrix is required to evaluate assay parameters, such as LOD, sensitivity (probability of detection), and specificity (*see* glossary for definitions). The sensitivity and specificity of the assay are experimentally determined using a set of target (inclusivity) strains, near-neighbor (exclusivity) strains, and matrix-relevant (background) organisms. Assay performance also needs to be measured in assay-specific matrices (i.e., blood, stool, water, soil, etc.). Often, assays are computationally designed using a set of available genomic/gene sequences at that time and then experimentally validated for signature presence in all available samples of the target organism (inclusivity panel) and validated for signature absence in many other samples that do not contain the target (exclusivity panel and matrix panel). In an ideal scenario, a laboratory routinely engaged in assay development could complete this process within 6 to 12 months.

**Figure 1.**
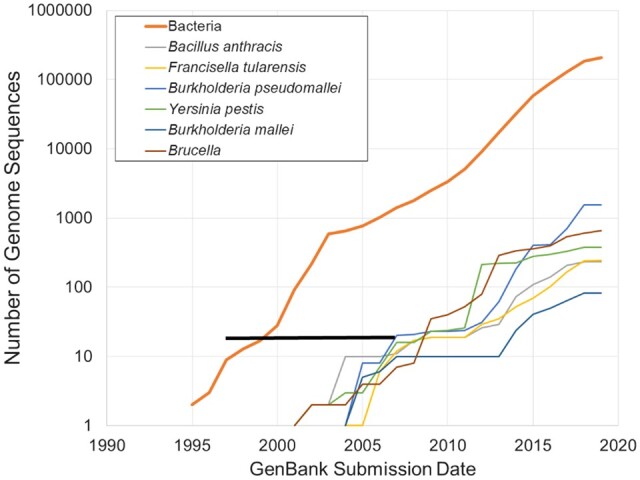
Availability of whole genome sequences for representative bacteria. Black bar represents the assay design time frame.

Detection assays are typically designed using all sequences available at that time. Many of the biodefense assays were designed and tested at least a decade ago when available sequences were limited. Thanks to recent advances in modern sequencing technologies, there is a sharp increase in the availability of whole genome sequences (Figure 1). Hence, these older assays have the potential to fail if evaluated against currently available sequences.

Moreover, publicly available sequence databases (e.g., GenBank) typically contain only a small fraction of naturally occurring sequence diversity. As a result, detection assays are vulnerable to “overfitting”: correctly differentiating known (i.e., sequenced) targets and nontargets but failing to detect novel target variants or falsely detecting novel nontargets.

Knowledge of the true genetic diversity is limited for some biodefense agents and their near neighbors, as often only several geographical and temporal representatives are fully characterized while other geographic locations have been ignored or significantly undersampled and hence are under-represented. In addition, while some agents, such as the bacterium *Bacillus anthracis*, are monomorphic (i.e., highly conserved), other agents, especially RNA viruses, are very diverse [e.g., Lymphocytic choriomeningitis virus (LCMV), Lassa virus, and Crimean-Congo hemorrhagic fever virus (CCHFV)]. In general, detection assays targeting highly conserved targets tend to fail due to unsequenced near-neighbor cross-reactivity, while assays targeting diverse targets tend to fail due to false negatives against unsequenced target variants.

While the recent revolution in next-generation sequencing technologies combined with decreasing sequencing costs has increased knowledge of population genomic structure, the capability for laboratory-based evaluation of newly sequenced strains has not kept pace. In this scenario, replacing or redesigning older assays to incorporate new knowledge of the target genomic landscape is critical. However, wet lab testing may not be feasible due to limitations on the timely availability of samples/strains. This problem is further exacerbated by policy decisions, such as the 2015 Department of Defense (DoD) moratorium that decreased access to live/inactivated biodefense pathogens for various applications, including assay development and validation (1).

### 1.1 Additional Considerations with the Status Quo Testing of Assays Against Inclusivity/Exclusivity Panels

The AOAC Stakeholder Panel on Agent Detection Assays (SPADA) inclusivity/exclusivity panels for the biodefense-relevant bacterial pathogens, such as *Bacillus anthracis*, *Yersinia pestis*, *Brucella suis*, *Burkholderia mallei, Burkholderia pseudomallei,* and *Francisella tularensis*, comprise a total of approximately 100 strains. These strains are used to validate the inclusivity/exclusivity criteria for the respective detection assays. Most of the inclusivity strains, and some exclusivity strains, are considered Biosafety Level 3 (BSL3) agents and, as a result, are limited to laboratories that are registered and certified for such work. Moreover, extensive laboratory testing adds cost and time to the assay development effort.

**Figure 2.**
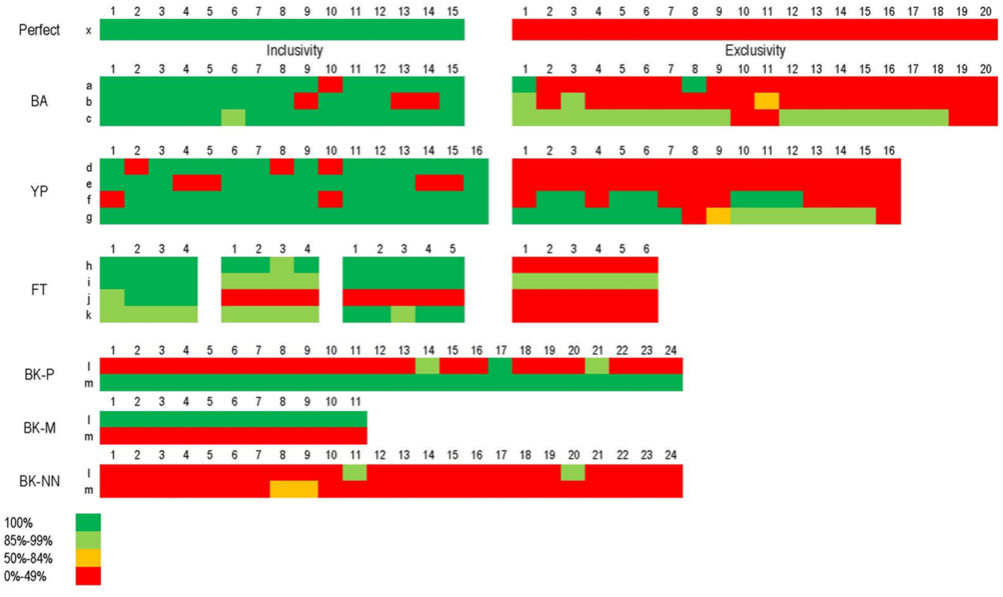
Signature sequence identities of the target sequences for inclusivity/exclusivity panel strains (SPADA panels). Perfect (x) refers to a hypothetical assay; BA: *Bacillus anthracis*, YP: *Yersinia pestis*, FT: *Francisella tularensis*; BK-P: *Burkholderia pseudomallei*, BK-M: *Burkholderia mallei*, BK-NN: *Burkholderia* near neighbors. Representative data set depicting the heat map of amplicon percentage identity in various whole genome sequences of bacterial strains used in inclusivity and exclusivity testing of molecular assays. Strains are numbered as columns from 1 up to 24. Each row (indicated by lower case letters) represents a given assay.

Many whole genome sequences of these bacterial strains are available now (2–6), which allows the *in silico* evaluation of assays. An example set of assays developed prior to the next-generation sequencing revolution with representative analyses is illustrated in Figure 2. As expected, the majority of the evaluated assay signatures had perfect sequence matches to the target inclusivity genome sequences, and much less (0 to 40%) sequence identity to the exclusivity panel genome sequences. However, for all the assays evaluated, there was no “perfect” assay (i.e., no false positives and no false negatives). Some assays were computationally predicted to have both false negatives (e.g., *Bacillus anthracis* assay 1 against strain 10 in the inclusivity panel) and false positives (e.g., *Bacillus anthracis* assay 1 against strain 8 in the exclusivity panel). Many of these predicted assay failures correspond to expected deviations based on the genotypes of these strains. There are other assays that simply fail the inclusivity and/or exclusivity criteria (e.g., *Bacillus anthracis* assay 7 or *Yersinia pestis* assay 15) and are therefore not reliable diagnostics due to low specificity. However, given the high conservation of the assay signatures to the target strains in the inclusivity panel and their low conservation in the exclusivity panel, the “brute force” testing of all available strains is not cost effective. As described below, a cost-effective selection of inclusivity and exclusivity strains for testing can be guided by *in silico* analyses and *in silico* PCR testing (section 2.2).

### 1.2 Additional Considerations with the Availability of Inclusivity/Exclusivity Panel Reference Materials

**Figure 3.**
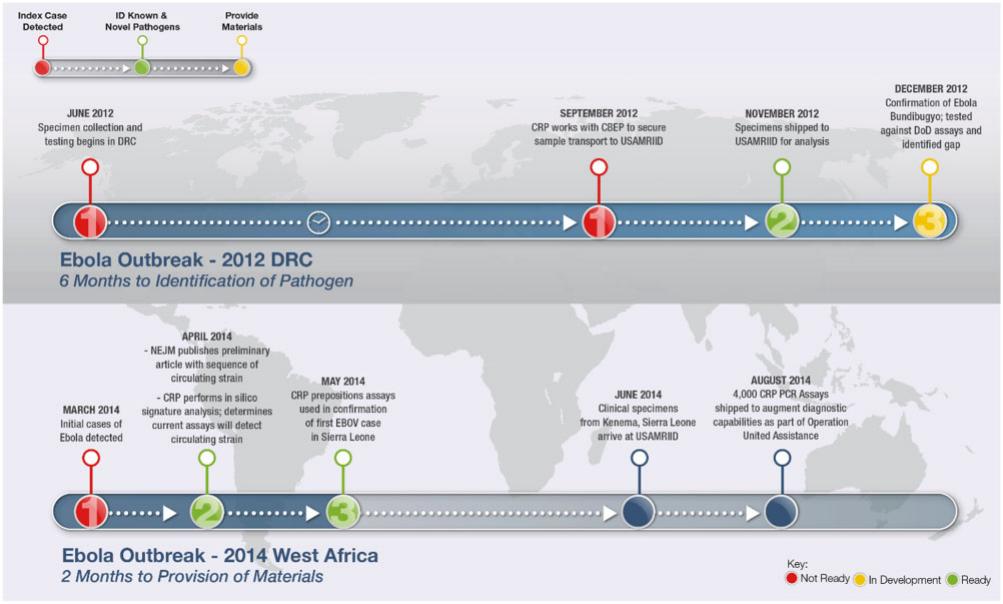
(Courtesy: Kristin Jones Maia, DBPAO internal brief). Examples of the timeline for obtaining Ebola reference materials from Africa. A delay in obtaining pathogen reference materials may hamper the discovery of signature erosion and the follow-on development of new or ‘old and improved’ detection assays, thereby delaying an effective assay from reaching the field in a timely manner. ID- Identity, DRC- Democratic Republic of Congo, CRP- Critical Reagents Program, CBEP- Cooperative Biological Engagement Program, USAMRIID- United States Army Medical Research Institute of Infectious Diseases, DoD- Department of Defense, NEJM- New England Journal of Medicine, EBOV- Zaire Ebola Virus.

A 2015 DoD moratorium on Biological Select Agents and Toxins (BSAT) work has constrained the transfer of select agents between labs for testing during assay development (1). In addition, obtaining reference materials from disease outbreaks and foreign locations has become increasingly difficult due to geopolitical sensitivities and the length of time involved in establishing Inter Agency Agreements. For example, in the 2012 Ebola outbreak, there was a delay of over 6 months in obtaining reference materials from Africa for evaluating assay performance (Figure 3). Thus, it took 6 months to realize that there was a gap in detection in that the then-available Bundibugyo assay failed against the outbreak strain. Due to this delay in obtaining reference material or whole genome sequence information, an effective redesigned assay could not be developed in a timely manner. In the 2014 outbreak, the availability of whole genome sequences within a short time (≈1 month) after the identification of the index case (7) allowed *in silico* evaluation of the existing assay’s efficacy in detecting the new strain.

Hence, there is a heightened impetus for developing robust *in silico* methods and synthetic biology approaches to reduce the need for live/inactivated pathogen samples. While *in silico* assay design approaches cannot circumvent the need for experimental testing against actual pathogens in relevant biological samples, *in silico* methods can be used to direct the experimental testing to those isolates that are most likely to demonstrate assay failure (due to false positives or false negatives). Computational prioritization of testing has the potential to streamline efficiency by providing robust characterization while minimizing the time, cost, and sample resources consumed. While this document is not intended to promote specific applications or software, it is intended to describe recommendations and guidelines for modern *in silico* assay design and evaluation.

## 2 Assay Development Process: Traditional (Low Throughput) vs Modern (High Throughput)

**Figure 4.**
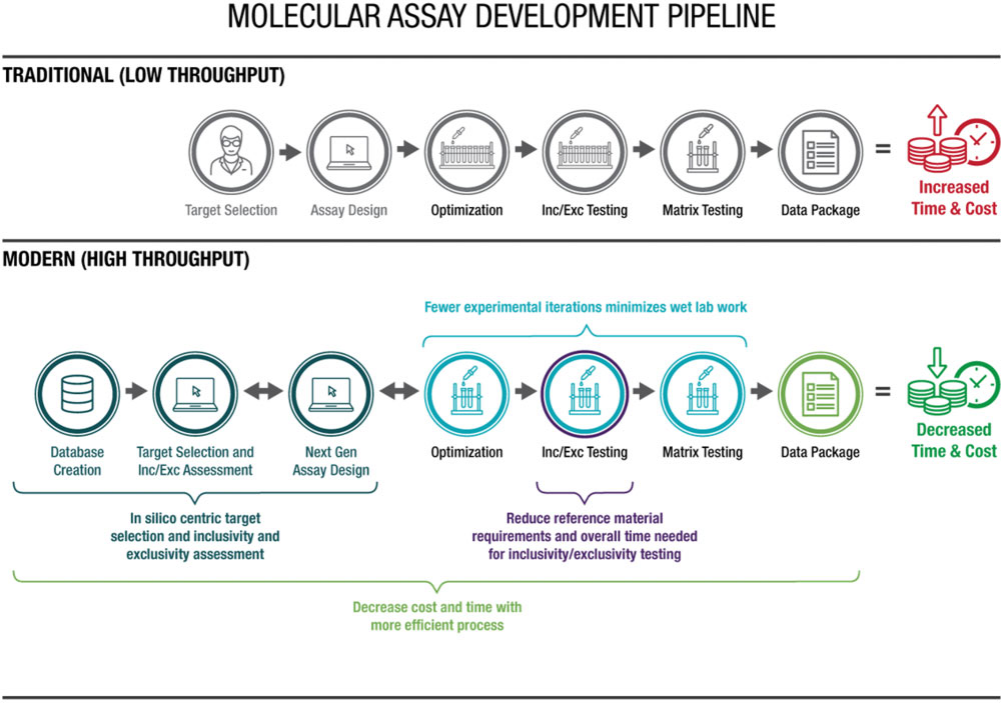
Traditional vs. modern assay development pipeline. Inc/Exc: Inclusivity/Exclusivity.

Traditional and modern assay development processes are illustrated in Figure 4. Apart from the initial assay design step, the traditional approach is centered on laboratory wet lab testing. The key objectives of the modern process are extensive use of *in silico* analyses of whole genome sequences to (1) guide and minimize the number of experimental iterations, (2) minimize the inclusivity, exclusivity and environmental panel wet lab testing, (3) address limitations on obtaining reference materials, and ultimately (4) cut down cost and time while improving assay performance. Essentially, the modern approach is data-driven and requires (*a*) establishing well-curated sequence databases, and (*b*) using state-of-the-art assay design algorithms to evaluate assay designs and rank assays prior to wet lab testing (detailed below). This approach reduces the number of experimental iterations compared to the traditional approach. The following sections compare and contrast the various steps of the two approaches.

## 3 Assay Design

### 3.1 Target Selection: User Defined vs Unbiased (*In Silico*)

Traditionally, assay target selection has been an ill-defined process that is strongly influenced by the preferences and experience of individual assay designers. Often, assay targets are selected from lab-specific research interests on specific genes of given pathogens or from known (literature-based), or suspected, virulence factor genes. The resulting assays are then screened (either computationally or “by eye”) for inclusivity and exclusivity using hand-selected sequences. The resulting assays are also often broadly screened computationally against all known sequences [e.g., by using Basic Local Alignment Search Tool (*BLAST*)] to identify potential false-positive organisms, but this exhaustive approach is not optimal because it detects many hits that are not relevant to sequences that might be found in relevant sample matrices (e.g., body fluids or soil). In other words, screening primers against all known sequences is overly restrictive to proper design. In contrast, a modern approach uses an unbiased search of all available sequence data for the organism of interest to identify potential targets and then validates those targets/genes against well-defined inclusivity, exclusivity, and environmental background sequence panels (e.g., SPADA environmental panel list of organisms).

### 3.2 Traditional Primer Design Paradigm

**Figure 5.**
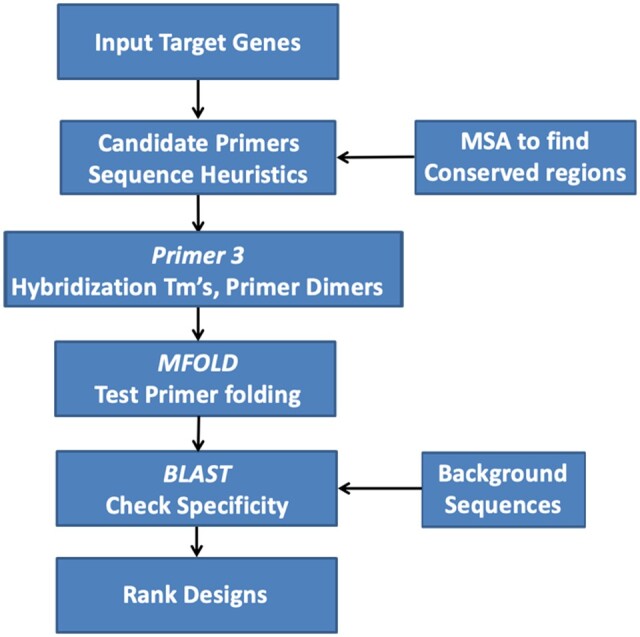
Traditional primer design approach. MSA: Multiple sequence alignment. The software tools depicted are only exemplar suggestions and not an endorsement of specific tools. Any other software with an equivalent functionality can also be used for producing similar outputs.

Primer design is a critical aspect in the development of diagnostic assays that has been relatively neglected compared to other parts of the process, such as instrumentation, enzymes, buffer additives, and data analysis. However, high-quality primer design offers a tremendous opportunity to improve diagnostic performance (i.e., sensitivity, specificity, and LOD), as well as reduce the development time and cost. Figure 5 shows a traditional primer design approach. Such a design pipeline brings together numerous tools that work well for their intended uses, but were not specifically optimized to be used together for primer design. As a result of the deficiencies of such traditional approaches, developing a high-performing assay requires extensive experimentation with numerous cycles of redesign and testing and even after significant financial investment, the resulting assays are often fragile and prone to failure (8). Below, modern methods are recommended for each step in the assay development pipeline. These methods are database-driven, apply physical chemistry modeling, and utilize modern design algorithms and computational resources to overcome some of the weaknesses associated with the traditional approach.

### 3.3 Modern Primer Design Paradigm

#### 3.3.1 Modern sequence databases

Perhaps the most important new contribution to the field of PCR is the availability of modern sequence databases—they are a treasure trove that can be used to improve the design of the PCR assay to maximize coverage (i.e., the number of variants that are efficiently amplified by a given PCR reaction) and also guide the testing of a PCR assay by identifying potential false positives or false negatives (e.g., due to sequence variations at primer and probe sites). When designing a PCR assay, it is helpful to first collect sets of sequences that represent the inclusivity, exclusivity, and background panels (*see* glossary for definitions). In addition to the generalized databases, such as National Center for Biotechnology Information (NCBI) GenBank, European Molecular Biology Laboratory-European Bioinformatics Institute (EMBL-EBI), and the DNA Data Bank of Japan (DDBJ), there are a variety of invaluable curated pathogen genome databases, such as the Virus Pathogen Database and Analysis Resource (ViPR), NCBI viral genomes, Los Alamos Hemorrhagic Fever Viruses Database, and Virulence Factor Database (http://www.mgc.ac.cn/VFs/main.htm). We also recommend using primer design software tools that utilize such databases as an integral part of their design, such as *BioVelocity* (9) and *PanelPlex* (DNA Software, Inc.), to simplify the task of database management.

#### 3.3.2 Inclusivity databases

Important considerations for genome databases include the issues of sequence quality, missing data and metadata errors. Sequence quality refers to the likelihood that, at each position in a genome sequence, the given nucleotide is correctly specified. Sequence quality is impacted by a number of factors, including unnatural mutations in lab-adapted strains, sequencing errors, mis-assembly, and experimental contamination. Missing data can include genomes for which only a portion of the genome sequence is available (usually the product of amplicon sequencing or bacterial draft sequencing), as well as sequences that contain unknown or ambiguous nucleotides [typically represented by the International Union of Pure and Applied Chemistry (IUPAC) ambiguity codes]. Finally, metadata errors are errors in sequence-associated information (like taxonomic labels, clinical severity, and geographic origin) or incomplete metadata that can lead to a sequence being incorrectly included in, or excluded from, the inclusivity data.

It is recommended to include only high-quality sequences in the inclusivity database, since including poorly determined sequences can effectively reduce the number of conserved signatures regions in a set of target genomes. Use of partial sequences in the inclusivity can cause assay design algorithms to ignore otherwise promising regions and introduce artificial design constraints, thereby compromising design quality by introducing bias into the signature regions (e.g., due to the number of times partial sequences are present, rather than focusing on regions that are actually most conserved). Use of poor-quality sequences that contain deletions or inserted sequences can result in assays that detect “phantom” sequences that do not exist in nature.

The ideal case occurs when the inclusivity database fully represents the diversity of extant natural (or engineered) viral pathogens with high-quality, full-length genomes (e.g., Ebola, HIV, and Influenza A viruses). The availability of low-cost sequencing methods has made such high-quality genomes more common, though often such a ready-made, up-to-date collection does not exist. Then, it is incumbent on the assay developer to gather all available sequences into a curated inclusivity database taking the sequence quality into consideration (*see* above). Some viruses have highly variable genomes [e.g., the human rhino viruses (HRV types A and B), human papilloma viruses (HPV), LCMV, Lassa virus, and CCHFV]. For such highly variable viruses, utilizing full-length genomes (and removing partial sequences) is of paramount importance for high-quality PCR design.

Alternatively, there are some viruses (e.g., Marburg virus subtypes Ci67, Musoke, and RAVN) where only a few examples have been fully sequenced to date. Such cases occur with newly emerging infectious diseases or diseases that have sparked little research interest. For these cases, utilizing only the few full-length genomes would result in “over-fitting” wherein many regions appear to be conserved, but in fact deeper sequencing would show that many of those regions are not appropriate for primer design. It is advantageous therefore to include both full-length as well as partial and incomplete genomes in these inclusivity data sets. However, as most assay design methods attempt to maximize the number of inclusivity sequences detected with the smallest number of assays, including *unmodified* partial sequences will force assays to cover the regions that have been sequenced most often, rather than focusing on the regions of the genome that are actually most conserved. While this can be a good strategy when dealing with highly variable genomes for which strain diversity is better represented by available amplicon sequences than available whole genome sequences, it is a poor strategy if the available amplicon sequences are generated from a hypervariable region or a region that is perfectly conserved in near neighbors. An alternate strategy is to “fill in” and “extend” missing sequence data by interpolating and extrapolating partial and incomplete sequences (10).

Bacteria also present challenges for many design algorithms since they usually have circular genomes without a defined starting point, and they code for proteins on both strands. As a result, different sequencing labs can publish the genomes with different strands and/or starting points. Thus, it is useful to perform work up front to include the same strand in the inclusivity database for all members of the set. Bacteria also present challenges due to their genomic DNA size that is roughly 100 to 1000 times larger than that of viruses, thereby placing demands on computational CPU (Central Processing Unit) and memory resources for signature analysis algorithms (below, we describe efficient k-mer algorithms that are capable of handling bacterial genomes). For bacterial inclusivity databases, it is recommended that partial genomes be segregated into a separate database from the full-length genomes. Partial genomes can then be avoided for purposes of design but later included in testing for coverage with an algorithm such as *Primer-BLAST* or *ThermoBLAST* using a combined database of full-length and partial genomes. In instances where there is an abundance of sequencing for a particular gene (e.g., 16S ribosomal RNA, a particular conserved virulence factor, or a toxin gene) from an organism, it is important to include in the inclusivity database only sequences (complete or partial) that contain that gene of interest.

**Figure 6.**
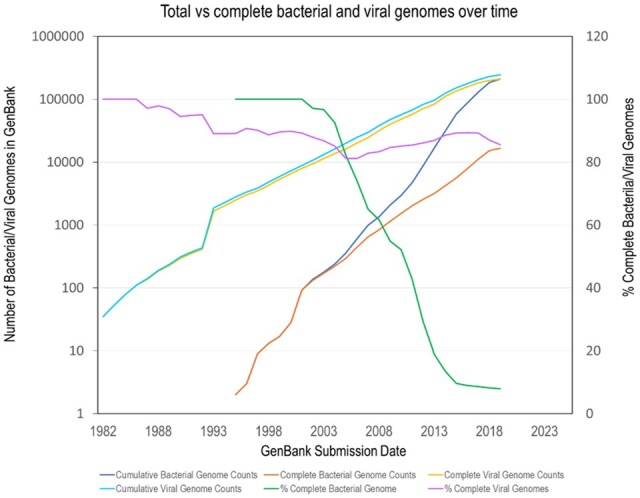
Plot showing the total number of draft and complete bacterial genomes in GenBank and the percentage that are complete as a function of year. These plots were made by parsing the “prokaryotes.txt <ftp://ftp.ncbi.nlm.nih.gov/genomes/GENOME_REPORTS/>” that NCBI provides as an inventory of all bacterial genomes. Data accessed on May 24, 2019. For viral genomes, the plots are based on the viral genome summary table: https://www.ncbi.nlm.nih.gov/labs/virus/vssi/#/virus?VirusLineage_ss=Viruses,%20taxid:10239&SeqType_s=Nucleotide. The data were accessed on August 22, 2019.

The number of bacterial and viral genomes in GenBank continues to climb (Figure 6). The low cost of generating short-read sequences using next-generation sequencing has led to increased production of draft microbial genomes consisting of multiple contigs. Although complete finished genomes can be generated by combining these contigs with long-read sequences obtained from platforms such as PacBio or Oxford Nanopore with nominal additional cost, there is a decline over time in the percentage of available genomes that are complete finished genomes versus draft genomes (Figure 6). At the same time, perhaps, with smaller genomes (e.g., viruses) there is an increase in percentage of full-length genomes over time. Along with the expected exponential increase in the size of databases will be a growing demand on the computational resources to handle such larger databases.

#### 3.3.3 Exclusivity and background databases

For the purposes of checking for false-positive amplifications, it is useful to construct exclusivity and environmental background databases. For computational efficiency, we recommend populating the exclusivity database with near-neighbor sequences (i.e., organisms that are phylogenetically distinct but closely related to those in the inclusivity data set). All other distantly related organisms that may be present in the sample matrix and might cause false positives can be placed into the background database. Further, we recommend that the background database consists of unrelated genomes that cover the normal flora that can be present in a clinical matrix or other potential interfering microorganism contaminants (e.g., nontarget soil microorganisms in an environmental sample, etc.). For both the exclusivity and background databases, sequence quality is generally not an issue and it is recommended to include partial sequences as well as complete genomes. A common practice is to check primers for reactivity with all known organisms [such as the GenBank nonredundant (nr) or nucleotide (nt) databases] using a program such as *BLAST* or *primer BLAST* to detect all off-target hits and amplicons. However, it is *not recommended to use such exhaustive databases during the design stage* because the nt and nr databases contain many sequences that have no possibility of ever occurring in the sample matrix and thus, including such exhaustive sequences would provide restrictive design constraints that are not valid and could result in a suboptimal design.

#### 3.3.4 Target region selection

Traditionally, the first step in design is to find a region of the pathogen genome that is conserved among variants of a given target. A multiple-sequence-alignment (MSA) algorithm (e.g., *CLUSAL, T-COFFEE, MAFFT,* or *MUSCLE*) is the traditional approach to identify such conserved regions. However, MSA algorithms do not scale well (in terms of CPU and memory) with either large numbers of sequences or with long sequence lengths. Even with modern cloud computing resources, computing a large MSA can be intractable. In addition, pathogen DNA and RNA sequences vary significantly in their number of bases, substitutions, insertions, and deletions. When combined with the low complexity of nucleic acids (i.e., only 4 bases for nucleic acids vs 20 amino acids for proteins), it is particularly difficult to get the high-quality alignments that are required to deduce the desired conserved regions. These limitations make it essentially impossible to apply an MSA to large collections of bacterial genomes or highly variable viral genomes (e.g., LCMV, CCHFV, Lassa virus, HPV, and HRV). Sequence alignments of the final design region, however, are helpful for displaying the variations present and provide a helpful reality check after a design region is discovered with a k-mer approach. Thus, we do recommend using an MSA that is restricted to the design region of interest, but not for the entire genome.

A superior approach for determining the optimal design region(s) is to analyze targets using k-mers (i.e., substrings of length k, usually 14–25, depending on the application; the rationale is described in references 8, 9, and 11). Such k-mer algorithms are computationally efficient for large databases and long sequences and can be applied to databases of pathogenic viruses and bacteria. An optimal design region from a pathogen would show *high* conservation among the variants of the desired target (e.g., clinical isolates of a pathogen) and show a *lack* of conservation to near-neighbor organisms or to contaminating organisms that could cause false positives. Thus, we recommend the use of k-mer algorithms to analyze inclusivity and exclusivity genome databases to determine optimal locations of signature design regions. One such algorithm is described in the literature by Yuriy Fofanov’s group (8) and applied to the development of an assay for the 2001 pandemic H1N1 influenza A. Such a k-mer algorithm is also available in the commercial *PanelPlex-Consensus* program (DNA Software, Inc., Ann Arbor, MI, USA). Other alternative approaches include Uniquemer (11), *BioVelocity* (9), or Core/pan genome analyses to identify unique genes that can be assay targets (12, 13). In all these approaches, the key first step is to create the inclusivity, exclusivity, and environmental background panels.

#### 3.3.5 Physical chemistry modeling

Predicting the strength of primer hybridization is critical for assay design (14). Most design programs (such as those available from many commercial oligonucleotide synthesis vendors) utilize nearest-neighbor thermodynamic rules to compute the 2-state ΔG°T, ΔH°, ΔS° (these are the standard state change in Gibbs free energy at temperature T, standard state enthalpy change, and standard state entropy change, respectively), and melting temperature, Tm, (15). In performing such hybridization predictions, most programs rely on the 2-state Tm to determine hybridization quality. Tm is intuitively useful because it is the temperature at which 50% of the target is bound by the oligonucleotide and 50% is unbound. However, Tm does not indicate the amount of hybridization at the desired annealing temperature for primers or at the extension temperature for TaqMan probes. A common misconception is that the best way to design primers is to match their Tms (14). This procedure is suboptimal, however, for two reasons: (1) even if the Tms are matched, the binding curves have different slopes (due to different ΔH° values) and thus different amounts bound at the annealing temperature; and (2) the 2-state Tm does not capture the competing unimolecular secondary structures (14). Primer and target unimolecular secondary structure can be predicted using dynamic programming algorithms such as MFOLD (16), RNAStructure (17), or OMP (14). Rather than focusing on Tm-based metrics, it is recommended to use software that focuses on solving the competing equilibrium for the actual amount bound at the desired temperature. The algorithms should try a wide variety of primer/probe lengths so that G-C rich targets will use shorter primers/probes to achieve a particular amount bound, while A-T rich targets will naturally select longer primers/probes to achieve a similar amount bound. Computation of the amount bound is best accomplished using a multistate coupled equilibrium model (14, 18). In addition to computing bimolecular hybridization and competing unimolecular folding, it is useful to check sets of primers to ensure that they do not form primer-dimer species involving the 3’-ends of the primers. This can be predicted with programs such as AutoDimer (19) and *ThermoBLAST* (14). There are also a variety of experimental approaches for eliminating primer-dimers (20, 21).

#### 3.3.6 Checking for specificity and coverage

Traditionally, the *BLAST* algorithm (22) is used to scan primer candidates against a database of genomes to determine if the primer hybridization is specific. *BLAST* was developed to deduce sequence similarity using evolutionary scoring, and *BLAST* is outstanding for such applications. However, for primer design, sequence *similarity* is not actually the metric that matters most. Instead, the quality of the *complementarity* to a primer is the scoring criteria that matters for primer design. A better approach is to use thermodynamic scoring (i.e., hybridization ΔG°_T_ or the amount bound from the multi-state coupled equilibrium model). Such thermodynamic scoring properly accounts for sequence and length as well as the effects of strand concentrations, salt conditions, and temperature. Examples of programs that perform scanning of oligonucleotides against genome databases are *ThermoBLAST* (14), *Primer-BLAST* (22), and *Thermonucleotide BLAST* (23). A significant advantage of these programs over *BLAST* is their ability to not only find thermodynamically stable hits, but also to evaluate if the hits are extensible by a polymerase (i.e., matched pairing at the 3’-ends of the primers) and determine if pairs of primers are pointing in opposite directions and within some length window (e.g., less than 1000 nucleotides) so that all possible amplicons are detected (e.g., *ThermoBLAST*). Notably, the various programs are not all equally proficient at detecting all amplicons (e.g., some programs, such as *Primer-BLAST*, do not detect mismatched hybridization very well).

#### 3.3.7 Probe design

Most instrumentation for detecting a PCR reaction requires the use of a fluorescent moiety. Addition of intercalating dyes, such as SYBR Green (and many others), is useful for testing the quality of primers for formation of a proper amplification curve (i.e., a single transition with an “S”-shaped saturation curve, appropriate Cq value, and curve amplitude) in the presence of target genomic DNA and performing no-template controls. However, such intercalating dyes detect all amplification products (i.e., both the desired amplicon and off-target amplicons) and thus dye-based methods are notorious for false positives. Therefore, the use of dye-based detection is not recommended for diagnostic assays. Further confirmation that the observed amplicon is the bona fide target of interest requires independent amplicon sequencing (e.g., Sanger sequencing method). For diagnostic assays, the use of an oligonucleotide probe (e.g., TaqMan, molecular beacon, or capture probe) provides an extra level of specificity in that only amplicons that bind to the probe are detected (and most such probe-binding amplicons are indeed the desired target sequence). Comparison of the dye-based detection with the oligonucleotide probe-based detection can provide invaluable confirmation that an assay is performing correctly. The thermodynamic design principles for oligonucleotide probes have been reviewed previously (14) and will not be covered further here. Modified probes such as minor groove binders (MGB) and locked nucleic acids (LNA) bind more tightly and specifically to their intended targets so that shorter probe sequences can be used compared to probes that contain only natural nucleotides. Shorter modified probes can be particularly helpful for highly variable viruses and bacteria that do not have a large signature region available. However, a drawback of such MGB and LNA probes is that they can fail to bind to new variants of the target that contain mismatches, thereby making such modified probes more prone to signature erosion. If one intends to use modified probes, then acquiring a comprehensive inclusivity database that captures the breadth of variation is an essential prerequisite.

#### 3.3.8 Ranking the design results

A critical part of primer design is the ranking of the candidate designs using some sort of scoring equation. Unfortunately, there is no agreed upon “currency” for goodness of primer performance. Instead there are many metrics that have vastly different units, such as free-energy differences for folding and hybridization, amount bound, amplicon folding, target conservation, off-target hybridization, primer dimerization, primer-amplicon cross-hybridization, and a long list of nonthermodynamic rules [G-quartets, sequence complexity (or information entropy), amplicon length, etc.]. It is still something of an art to combine all of these disparate scoring terms into one big equation and produce a result meaningful to a user (such as a final score that ranges from 1 to 100). In addition, there is no agreement in the community as to what the relative weighting of different scoring terms should be. For this reason, it is recommended to use software that exposes the scoring equation and the weights used for each scoring term (e.g., *PanelPlex* provides a detailed description of the scoring). Transparency by software vendors regarding their scoring methods will give users the ability to change the scoring weights and also to be more informed about what the modeling is and is not accounting for. In the future when training and validation data sets become available as described in Metrology (section 5.0), the scoring terms and weights can be optimized by solving for the optimal weighting terms. These datasets will also support the ability to evaluate the predictive quality of software from different commercial and noncommercial sources.

#### 3.3.9 Combining all of the recommendations into a coherent design pipeline

**Figure 7.**
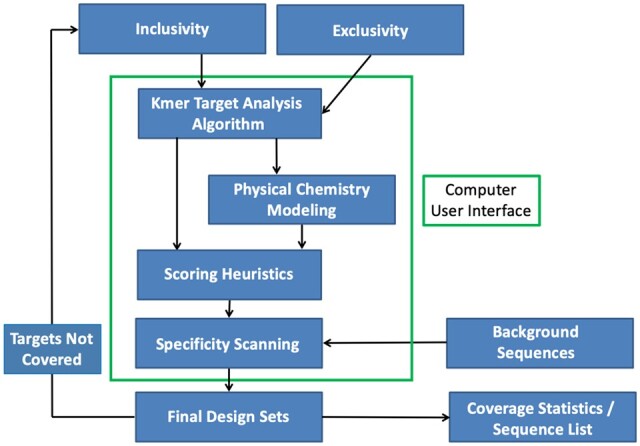
Modern Design Paradigm for Pathogen Detection by PCR.

In Figure 7, we provide an example of a design pipeline that includes the aspects of the modern approach described above. Foremost in this modern approach is the integrated use of sequence databases for inclusivity, exclusivity, and background. These are used in the k-mer-based target analysis algorithm as well as the thermodynamics-based scanning of oligonucleotide candidates to determine their coverage and specificity. There is also built-in physical chemistry modeling (e.g., dynamic programming algorithms such as *MFOLD* or *OMP*) to compute thermodynamics for unimolecular folding and bimolecular hybridization, and numerical methods for solving the multistate coupled equilibrium model to determine the amount bound. There is also the critical component for ranking the results using a weighted scoring equation. Lastly, Table 1 provides a summary of essential design criteria that should be included in a modern primer design pipeline. Improving upon these principles will require implementing the recommendations described in the metrology section below. Combining the designs for different single-plex reactions into a larger multiplexed format is discussed in the next section. In addition, below an iterative process is recommended for performing experimental validation to develop robust assays.

**Table 1.** Summary of recommendations for PCR primer design

Design stage	Item	Recommendation	Reason
Preparation for design	Inclusivity database	Database and literature research on target sequences	Consensus design to conserved regions
	Inclusivity database	Full-length genomes or genes	Fragments lead to design bias
	Exclusivity database	Near-neighbor sequences	Sequences likely to cause a false positive due to sequence relatedness or similar symptoms
	Background database	Gather contaminating genomes	Reduce false positives from human genome, human refseq, human microbiome, soil microbes, etc.
	Reaction conditions	Gather enzyme, buffer and salts, [NTPs], [Primers, Probes]	Needed for proper physical modeling and design
Software input	Run software	Save all user settings and input, scoring terms and weights	
	Save input file	Capture all software parameters	Allow for design to be reproduced, capture input for future meta-analysis using A.I.
	Run from input file	Software can be run from input file	Allow for design to be reproduced, reduce input errors in subsequent runs
Algorithm	k-mer analysis	Use to find signature regions using inclusivity and exclusivity databases	Find signature regions that are conserved in the inclusivity and not found in exclusivity
	Sequence alignments	Do not use for finding signature regions	Not applicable to long genomes and to many genomes
	Sequence alignments	Align design regions of all members of inclusivity	Use for reality check of consensus region only
	Physical chemistry modeling	Target secondary structure, primer hybridization, primer dimers	Naïve 2-state Tm is not sufficient
	Heuristic scoring	Scores for non-thermodynamic considerations	Examples: G-quartets, low complexity, amplicon length, amplicon folding, etc.
	Specificity scoring	Use thermodynamics-based scanning of primers against genomes	Check if primers are specific (PrimerBLAST, ThermoBLAST, ThermonucleotideBLAST)
	Coverage scoring	Determine how well primers cover all members of inclusivity	Determine mismatches and amount bound for all members of inclusivity
	Total scoring	Weighted scoring equation	Combine thermodynamic, heuristic, kmer, specificty, and other terms into a total score.
	Add positive control	Check for compatibility of singplexes with the control gene	Don’t want control to interfere with desired target(s)
	Multiplex primer dimer check	Check for all possible primer dimer interactions	input for multiplex algorithm
	Multiplex primer-amplicon check	Compute cross-hybridizations of all primer candidates against all amplicons	Compute cross-hybridizations of all primer candidates against all amplicons
	Multiplex primer-background check	Compute false amplicons of all primers against background database	Multiplex algorithm needs to minimize false amplicons to allow even amplification of all targets
	Multiplexing	*in silico* analysis and combinations of single-plexes	Determine optimum combination of singplexes that are mutually compatible with eachother
Experimental validation	Determine performance of single-plexes	Capture single-plex performance metrics	Capture single-plex performance, link to input file information, allow for future A.I. meta-analysis
Redesign	Redesign of failed single-plexes	Tools that allow for replacement of poor performing primers	Hold constant primers that work, redesign primers for targets that don’t work
	Redesign of failed mutliplexes	Tools that allow for replacement of poor performing primers	Hold constant primers that work, redesign primers for targets that don’t work
	Multiplexing	*In silico* analysis and combinations of single-plexes	Determine optimum combination of singplexes that are mutually compatible with eachother
Experimental validation	Determine performance of multiplex	Capture multiplexplex performance metrics	Capture multiplex performance, link to input file information, allow for future A.I. meta-analysis
Post-design analysis	Assay stewardship	Tools for analyzing existing assays using new target variants	Ensure that existing assays work on new target variants
	Final report	Software summary of primer/probe performance	Summary of final design: thermodynamic, heuristic scores, specificity, sequence alignment
	Metrology	Analyze input and performance metrics to determine best practices	validate and verify predefined standards for traceability, accuracy, reliability, and precision

#### 3.3.10 Multiplexing

Multiplexing involves performing numerous assays in the same reaction chamber. Multiplexing has the advantages of reducing the number of tests, thereby saving reagents, time, money, and also limiting the amount of sample needed. Such multiplexing can be as small as 2-plexes where the desired target is PCR amplified in the presence of an internal positive control (e.g., M13 bacteriophage or RNase P control) to much larger multiplexes where numerous pathogens are detected in the same reaction. The major challenge of multiplexing is to find sets of primers and probes that are “mutually compatible” under a given set of reaction conditions. “Mutually compatible” means that the primer sets amplify with similar efficiency, do not cross-hybridize to incorrect amplicons, do not form primer-dimers, and do not form false amplicons involving the matrix background. Designing the primers to amplify at similar rates is critical to ensuring that amplification of one or more targets does not overtake the reaction and consume all the reagents or bind to all of the enzyme. Uniform amplification efficiency can be achieved using the principles described above (physical chemistry modeling) to design primers that bind to thermodynamically exposed (i.e., unfolded) regions of the target. These designs should result in amplicons that do not have significant folding that can inhibit polymerase extension and primers that do not form competing hairpins. Experimental testing of candidate single-plexes to ensure that each one amplifies efficiently and does not give a false positive in the no-template control reaction is highly recommended before proceeding to multiplex testing. Minimizing the formation of primer-dimers is relatively easy to check computationally (19). However, the exponential explosion in the number of possible multiplex reactions makes it computationally intractable to use a brute-force approach to check all possible multiplex permutations for all possible artifacts that can occur (*see* below).

#### 3.3.11 Multiplexing is a complex system

Multiplex PCR is a complex system with many interacting variables (e.g., multiple primers, genomic DNA from the target(s) of interest and contaminating genomes from the sample matrix). Optimizing one set of primers can result in an unexpected interaction with another set of primers in the reaction to create unexpected false amplicons from the exclusivity and background databases or primer-amplicon cross-hybridization reactions. In addition, as the size of the multiplexed reaction grows larger, there is a combinatorial explosion in the permutations of possible multiplex reactions. Consider a 20-plex reaction with 10 primer design candidates (i.e., top-scoring single-plex designs) for each of the 20 targets: the number of possible multiplex reactions to consider is 10^20^. An iterative empirical approach can only assess a few reactions (without knowledge of the interacting variables) and thus samples a very limited amount of the sequence design space, almost always resulting in a suboptimal design and often complete failure. Even an iterative brute-force computational approach using high-performance computing that could test millions of combinations of multiplex sets will vastly under sample the number of possible reactions. For the 20-plex example with 10 candidate solutions, it would be computationally intractable to run thermodynamic scanning (e.g., *PrimerBLAST* or *ThermoBLAST*) for all 10^20^ possible multiplex permutations to ensure that false positives are minimized. Thus, a modern sophisticated algorithm is needed to solve the combinatorial explosion thoroughly and find the optimum multiplex design. The program *PanelPlex* uses a depth-first search with a pruning algorithm to accomplish the multiplexing design task and guarantees finding the optimum solution (or top N solutions) out of the entire space of possible multiplexes. We recommend using such multiplex optimization algorithms to greatly reduce the number of iterations required to discover a high-performing multiplex reaction. No modeling algorithm is perfect, and there are many variables that are unknown even in the most sophisticated design paradigm. However, using such sophisticated multiplex design should result in many fewer design and experiment iterations and vastly superior assay performance.

## 4 Metrology for *In Silico* Analysis

Metrology is the science of measurement and serves an important, but often under-appreciated role in the development and validation of *in silico* PCR assay design methods. Measurement assurance concepts that help increase confidence and decrease uncertainty (the error associated with a result) for experimental data (24) can also be applied to *in silico* approaches.

### 4.1 Sources of Measurement Uncertainty

One of the key steps in PCR assay design is predicting the outcome of applying an assay to one or more DNA templates. While the information needed to define a PCR assay depends on the complexity of the computational model, relevant information can include (1) primer and probe oligo sequences and concentrations; (2) template sequences and concentrations; (3) salt concentrations; (4) thermocycling times and temperatures; (5) nucleotide concentrations; (6) polymerase concentration and properties (i.e., nucleotide extension rate); and (7) buffer composition. All these parameters can affect the final outcome and therefore contribute to the uncertainty that is the error associated with the model prediction.

However, it is often challenging to obtain accurate, quantitative measurements for many of these parameters in practice. Some information, like polymerase properties and the buffer composition, may be trade secrets that are not publicly shared (although some can be inferred or measured). Other information, such as template concentration, are often measured in ways that make biological sense (e.g., plaque forming units/mL, colony forming units/mL, etc.) but cannot easily be converted into the units of molarity required for modelling PCR with chemical kinetics or equilibrium thermodynamics. In the absence of accurate input data, unknown model parameters must be determined by fitting to measurements of PCR experiments. Note that models are not restricted to “physical” parameters (e.g., reaction rates and concentrations), but also include heuristic parameters such as sequence complexity and qualitative rules for primer design, e.g., avoiding runs of guanines; providing bonuses for ending primers with “A” to improve specificity; and similar patterns of sequences that should be avoided or enhanced even if the detailed physical reasons are not known. Continued progress in machine learning raises the possibility of training “black box” algorithms to predict PCR assay results based on a combination of physical process models (e.g., DNA hybridization) and experimental assay results. Regardless of how they are obtained, predicted PCR results should have an uncertainty associated with them, in order to help interpret results and make decisions about assay performance prior to moving to wet lab testing.

Another challenging aspect for metrology is defining the experimental quality of a given PCR reaction. Two widely used experimental metrics are the “quantification cycle,” Cq (25) and the PCR amplitude (26). Cq can be determined from the experimental PCR curve (e.g., by numerically determining the maximum second derivative point). For a given starting concentration of target DNA, the Cq is a metric for the amplification efficiency of the PCR. Primers that result in smaller Cq are generally considered to be superior to primers that result in higher Cq. Reactions that have poor hybridization due to weak 2-state thermodynamics, competing unimolecular folding, or consumption of primers due to off-target amplification would all result in higher Cq values. Reactions with a high Cq will have an inferior limit of detection if the number of PCR cycles is limited (this is why it is generally recommended to acquire no more than 45 cycles of PCR data; *see*Table 2). Another easily measured metric is the amplitude of the PCR curve (i.e., difference in fluorescence between the last cycle minus first cycle of the PCR reaction). High-performing PCR reactions have a large PCR amplitude and poor reactions will have smaller amplitudes due to poor performance of the probes or consumption of the primers due to off-target amplification reactions. However, there is currently no simple way to combine the Cq and amplitude metrics into a single score. Likewise, there is no good way to interpret the Cq and amplitudes to deduce what mechanism is causing a problem in a given PCR reaction. Lastly, both the Cq and the amplitude are dependent upon the concentrations of the primers and the probe, making it difficult to compare results across laboratories. As useful as the experimental Cq and amplitude are for characterizing the primer design quality, there is a need for the field to develop metrics that utilize all the information in the shape of a real-time PCR curve. A start in this direction is modeling the kinetics of the PCR reaction using the MAK2 model (27) and more recently in the program *CopyCount* (DNA Software, Inc.), which provides absolute quantification based upon full modeling of the PCR kinetics.

**Table 2.** Recommendations for PCR experiments

Item	Recommendation	Reason
Gather target sequences	Database and literature research on target sequences	Learn about target sequence variation, design PCR to conserved regions
Enzyme	Not HiFi, no 3'-exonuclease	3'-Exonuclease causes off-target amplification, PCR failure
PCR cycles	45	For 20 μL reaction, a single target molecule has Ct of about 38; using 45 cycles ensures detection
Denaturation temperature	First 3 cycles use 95°C and 20 s	Reduce delayed onset due to template re-annealing, lower Ct value observed
Denaturation temperature	Cycles 4–50 use 94°C for 5 s	Keep enzyme activity high
Dye-based detection	Good to evaluate if primers work	Test if primers work before ordering expensive fluorophore-labeled probes
Dye-based detection	Do not use for clinical testing	Often detect background amplification, causing false positives
No-template control	Run PCR without template DNA	Determine if “primer dimers” and other false amplicons are formed
Sanger sequencing	Perform sequencing on PCR reaction product	Verify that the amplicon product is indeed the correct target
Single-plex testing	Test all targets as single-plex before performing multiplex	If a reaction doesn’t work as single-plex, then it isn’t going to work in multiplex either
Positive control	Add to your analyte target	Verify that PCR is working, so that a negative for the target analyte is meaningful
Multiplex testing	Combine validated single-plexes into larger multiplexes	Verify that primers are compatible with each other
Synthetic target	Use synthetic gBlocks for initial testing	Cheap, nonpathogenic
Testing with patient samples	Use actual patient samples, known positives, and negatives	Determine sensitivity, specificity, and LOD
Gather assay information	Use MIQE standards	Ensure assay is reproducible and documented

### 4.2 Assessing Model Accuracy

In addition to the parameter measurements and inputs needed to develop and parameterize *in silico* PCR models, assessments of model accuracy are needed to compare and rank models so end users can evaluate the computational cost/accuracy tradeoff implicit in every model. While most computational models of PCR perform satisfactorily within the “well-behaved” limits of perfect match primers, short amplicons, no-template near neighbors, and single-plex PCR, many real-world applications do *not* fall into this category. Thus, a “ground truth” data set is needed to help determine model accuracy. The data set could be used to objectively evaluate algorithms from different research groups. The experimental, “ground truth” PCR data set would need to capture many details, including (*a*) target genome, (*b*) presence of contaminating organisms (determined through NGS sequencing), (*c*) enzyme and buffer compositions, (*d*) primer and probe concentrations, (*e*) composition of the amplicon products (by NGS sequencing to reveal the concentrations of the desired amplicon and off-target amplicons, primer dimers, etc.), and (*f*) composition of the PCR reaction at each cycle of PCR (e.g., real-time monitoring of the fluorescence, along with quantification of primer concentrations and enzyme activity). For this training data set (no publicly available data set), both the PCR inputs and outputs would be publicly revealed to enable the user community to improve and validate their *in silico* methods.

### 4.3 PCR data set in Support of Competitions to Spur Community Forward

Similar to the Critical Assessment of Protein Structure Prediction (CASP), there is a need for an open competition to assess the performance of different computational approaches for *in silico* PCR using experimental data. Competitions could provide a quantitative ranking of models by accuracy and spur the development of improved *in silico* models. In particular, to rigorously compare different *in silico* prediction methods, it would be important to experimentally explore “bad” real-time PCR assays, including (1) amplicons that are too long; (2) amplicons with strong secondary structure; (3) mismatches in both the forward and reverse primers; and (4) varying degrees of primer-hairpin and primer-dimer formation. For the training set, both the PCR inputs and outputs (described above in section 5.2 Assessing Model Accuracy) would be publicly revealed. For the validation sets, only the PCR inputs would be revealed. Outputs would be used for evaluation of PCR predictions from different research groups by independent referees. The final goal would be to quantitatively evaluate the performance of different *in silico* PCR methods (i.e., what did different models get right and wrong). To date there has not been an organized effort to provide funding to acquire the needed training and validation PCR data sets, develop an international committee to referee these contests, or recruit groups of scientists to engage in the contests. Successful completion of this effort would drive the field forward and have a dramatic effect on the quality of future PCR-based diagnostics.

## 5 Assay Development and Characterization

### 5.1 Assay Development

Once an optimal assay has been designed *in silico*, the next step is to assess the performance of the design in wet lab testing using appropriate templates and reagents. Given the increasing genomic diversity represented in genome databases resulting from improved sequencing technology, many diagnostic assays require the incorporation of degenerate primers and/or probes for efficacious species-specific detection. Quantitative assays require additional design considerations depending on the intended use of the assay. Specific genomic locations need to be targeted and methodologies employed if the desired readout is genome copies. The presence of mismatches in either the primer or probe binding locations can affect the PCR kinetics (i.e., the Cq value for a given target concentration) and thus impact the determination of target quantity. Amplification kinetics need to be determined for each new target variant to ensure accurate quantification. Best practices include sequencing the challenge stock to confirm the primers and probe are an exact match to the organism being quantified.

When possible, multiple assays are designed and tested, and poorly performing primer candidates are removed from further consideration or submitted for redesign or optimization of salt concentrations or thermocycling conditions before full-scale assay characterization. Usually a single live organism or inactivated organism strain or extracted/naked DNA/RNA (genomic material) or a surrogate with the assay target or synthetic assay target is used as template at this stage of the assay development process. Minimally, primer concentration optimization, especially if degenerate nucleotides are used, is needed to ensure optimal assay performance. Initial characterization studies can be done in a simple matrix such as water before conducting validation studies in the intended sample matrix. Analytical sensitivity includes determining the assay LOD, or the lowest concentration of organism that is reproducibly detected (e.g., when 58 of 60 test replicates are positive) (28, 29).

Analytical specificity involves both inclusivity and cross-reactivity (exclusivity) testing. For inclusivity testing, all the available strains or variants of the targeted organism are tested at levels above LOD. The strains selected for testing should represent any diversity observed within the assay target region. Synthetic nucleic acid could also be used to fill inclusivity gaps once the assay has been optimized and characterized using whole organism. Exclusivity testing should include other organisms located within the target genus, nucleic acid from the intended matrix (e.g., human whole blood), and any organisms that were identified during the initial amplicon *BLAST* analysis that have significant sequence identity. Such *BLAST*-identified targets that warrant further testing include organisms that have 90–95% sequence identity.

### 5.2 Assay Validation

While there are multiple methods for assay validation that can be used based on the eventual application, the approach described here and in Table 2 presents a summary of best practices for PCR assay validation for clinical use. Assay validation requires a comprehensive analysis of the test system (i.e., the sample to answer process including all steps such as extraction, testing, and analysis) to define the assay performance characteristics in the intended matrix, and the test system is only validated for that specific use. Both quantitative and qualitative assays require analytical sensitivity and specificity, precision, and accuracy testing with reproducibility via multiple replicates and testing runs being built into the testing design.

One comprehensive way of assessing analytical sensitivity in the intended matrix (29) is to conduct a preliminary LOD analysis with a confirmation of LOD, for example, incorporating at least five independent samples being run each day over 5 days. Assay precision reflects the repeatability of an assay when testing multiple aliquots of a sample (30). Validation expands the analytical specificity testing to include an assessment of interfering substances (e.g., heme from whole blood, humic substance from soil samples) on assay performance. Comparing assay linearity and LOD in the intended matrix to a simple matrix can help define that impact.

Reproducibility precision testing should incorporate as many potential variables as possible (e.g., different users, days, extraction instruments, real time PCR instrument etc.). Based on Clinical and Laboratory Standards Institute (CLSI) guidance (31), precision should involve testing three independent samples (at the LOD and 20% above and below the LOD) each day for 10 days. Accuracy defines how close the test result is to the actual value (32). In cases where a gold standard comparator is available, a direct comparison to the test assay can be made. In cases where such a comparator is not available, a mock clinical trial or recovery study can be performed (33). For example, 50 positive samples (1/3 at 1.5× LOD, 1/3 at a mid-relevant range, and 1/3 at a high relevant range) and 100 negative samples are tested over 5 days (34). Precision testing for quantitative assays is similar to qualitative assays except the samples generated are at a high concentration, a low concentration, and at the assay LOD (35).

In addition to the above analyses, quantitative assays require defining the reportable range of the assay, that is, the linear range of the standard curve where quantitative results can be accurately reported (36). Defining the lower limit of quantification (LLOQ) is done by testing 30 independent samples in duplicate at the lowest concentration within the reportable range and increasing the concentration until at least 58 of the 60 replicates are quantified with a predefined level of precision. The upper limit of quantification (ULOQ) can similarly be determined if needed. While only quantitative results within this range can be reported, one can also report qualitative results (positive or negative) that are outside this range (37).

### 5.3 Assay Stewardship—When New Viral or Bacterial Strains Are Discovered, Will the Existing Assay Work?

After an assay is designed and validated, it is relatively common for the scientific community to discover a new strain of a pathogen that has a mutation at a site that causes the assay to fail. This process is termed “signature erosion” (9). To date, there have not been any good methods for anticipating new mutations. One general recommendation is to design primers to be a little longer than needed for a perfect match. In the event that a single or double mutation occurs at a later time, the primers will still work as long as the new mutation does not occur at the 3’-end of either primer. In addition, there is a need to have software that can computationally test any new sequenced isolate against existing validated assays to predict if the new isolate is likely to be “covered” by the existing assay (i.e., if the existing assay is predicted to give an amplicon for the new isolate with high efficiency), or if the existing assay is likely to fail for the new isolate (i.e., the existing assay is predicted to give an amplicon for the new isolate with low efficiency). Even better would be for software to constantly monitor existing databases for new entries and to automatically alert a user or agency if a new isolate is “high risk” for assay failure. This capability would allow for real-time monitoring of assays to alert agencies in charge of protecting the public about potential assay failures. The agencies could then focus their ongoing validation efforts on those assays that have a high likelihood of failure rather than using their limited resources on potentially unnecessary redundant validations. Funding, development, and implementation of such an assay stewardship approach is highly recommended by the SPADA working group.

## 6 Regulatory Considerations for Nucleic Acid-Based Clinical Assays

*In silico* tools can be helpful for designing assays to address a wide range of clinical applications. *In silico* analysis approaches such as those outlined here, provide invaluable information for facilitating development of molecular assays and, in some cases, provide unique validation data in situations where wet testing is difficult. For example, *in silico* tools can identify potential cross-reactive species that can be further validated in analytical testing to determine the extent of cross-reactivity (i.e., concentration of microorganisms that can adversely impact assay performance) which provides information on assay specificity or identify known limitations (e.g., the extent to which closely related organisms can be differentiated). How *in silico* tools are incorporated into the design, development, and validation of an assay for clinical use depends on multiple factors that include the scope of the intended use, technology of the assay (e.g., IVD-nucleic acid-based test, etc.), test principal, and analyte(s) of interest. Collectively these also inform the type of regulatory submission a developer/sponsor would prepare for their device for FDA review prior to marketing.

The following is a general overview on the types of regulatory submissions a sponsor can prepare for a medical device, and is intended to serve as an introduction on how their device (i.e., a molecular assay or IVD that is within the scope of this document) can be regulated. FDA encourages sponsors to participate in the optional presubmission process if they have specific questions that they wish to be addressed by the agency on their medical device. Further information on the presubmission process is provided below.

### 6.1 Emergency Use Authorization Authority

Under section 564 of the Federal Food, Drug, and Cosmetic Act (FD&C Act) (38), the FDA Commissioner may authorize the emergency use of unapproved medical products or unapproved uses of approved medical products for certain emergency circumstances. Before FDA may issue an EUA, the Health and Human Services (HHS) Secretary must first declare that circumstances exist justifying the authorization based on one of four determinations made by either the Secretary of Homeland Security, Secretary of Defense, or Secretary of HHS of a material threat, an actual emergency, or a significant potential emergency involving a Chemical, Biological, Radiological, and Nuclear (CBRN) agent(s), or a disease or condition that may be attributable to such agent(s). More information can be found in the FDA finalized guidance “Emergency Use Authorization of Medical Products and Related Authorities” (39).

To help prepare for potential and current emergencies, FDA works with medical countermeasure (MCM) developers to prepare pre-EUA packages, when appropriate. A pre-EUA package contains data and information about the safety, quality, and efficacy of the product, its intended use under a future or current EUA, and information about the emergency or potential emergency situation. The pre-EUA process allows FDA technical subject matter experts to begin reviewing information and assist in the development of conditions of authorization, fact sheets, and other documentation needed for an EUA in advance of an emergency and also helps to facilitate completion of an EUA request during a current emergency declaration. Note that a pre-EUA can only transition to an EUA if there is a current applicable emergency declaration. Additional information on how to submit a pre-EUA for in vitro diagnostics to FDA can be found at the corresponding page at FDA’s website (40).

During the effective period of the HHS Secretary’s EUA declaration, FDA may authorize the introduction of a medical product into interstate commerce to be used in an emergency to diagnose, treat, or prevent serious or life-threatening diseases or conditions caused by CBRN threat agents when there are no adequate, approved, and available alternatives, provided that certain statutory criteria are met. When deciding whether to issue an EUA for a specific medical product, FDA determines whether the known and potential benefits of the product outweigh the known and potential risks based on the totality of the scientific evidence at the time of the EUA request. The EUA authority facilitates the availability and use of MCMs needed to prepare for and respond to public health emergencies. When an EUA declaration is terminated by the HHS Secretary, then any EUA(s) issued based on that declaration will no longer remain in effect and can therefore no longer be marketed.

### 6.2 FDA Regulatory Pathways

The overall objective for medical device manufacturers (i.e., assay developers) is to demonstrate reasonable assurance of safety and effectiveness to FDA prior to introducing a medical device into interstate commerce. The following is general information on FDA regulation of medical devices. This discussion is intended as an introduction to medical device development and regulatory review. FDA encourages sponsors to utilize the optional presubmission program in which they can submit requests for FDA feedback on any concerns (e.g., clinical trial design) or to discuss specifics that may pertain to the appropriate regulatory pathway for their device (41). As a rule, early discussions such as those through the pre-submission feedback mechanism facilitate agreement on items such as appropriate validation studies to support intended use claims, and additional transparency in the premarket review process.

#### 6.2.1 Device classification

The Medical Device Amendments (MDA) (Pub. L. 94-295) to the Federal FD&C Act (enacted in 1976) directed FDA to issue regulations that classify all devices that were in commercial distribution at that time into one of three regulatory control categories: Class I, II, or III, depending upon the degree of regulation necessary to provide reasonable assurance of their safety and effectiveness. The class into which a device is placed determines the requirements that a medical device manufacturer must meet prior to distributing a device in interstate commerce. According to section 513(a)(1) of the FD&C Act (21 U.S.C. §360c(a)(1)), the following are defined as the device classes:

- *Class I*.—Devices are subject to a comprehensive set of regulatory authorities called general controls that are applicable to all classes of devices.
- *Class II*.—Devices for which general controls, by themselves, are insufficient to provide reasonable assurance of the safety and effectiveness of the device, but for which there is sufficient information to establish special controls to provide such assurance.
- *Class III*.—Device for which general controls, by themselves, are insufficient and for which there is *insufficient* information to establish special controls to provide reasonable assurance of the safety and effectiveness of the device. Class III devices typically require premarket approval (PMA).

#### 6.2.2 510(k) program

Premarket notification is the process by which a new device (i.e., post-amendments device) is classified into one of the above listed device classes. A manufacturer who intends to market in the United States a Class I, II, or III device intended for human use, for which a PMA application is not required, must submit to FDA a premarket notification submission [often referred to as a 510(k)], unless the device is exempt from the 510(k) requirements of the FD&C Act and does not exceed the limitations of exemptions for each of the device classification regulations (sections 862.9–892.9 of 21 CFR Parts 862–892).

In a 510(k) submission, the Agency determines whether or not the device meets the criteria for market clearance. The Agency bases its decision on whether the device is substantially equivalent (SE) (i.e., as safe and as effective) to a legally marketed (predicate) device. Further information on the 510(k) program can be found in the Agency’s guidance “The 510(k) Program: Evaluating Substantial Equivalence in Premarket Notifications [510(k)]” (42). Additional information on the Agency’s potential actions from a 510(k) review and the impact on review durations can be found in the guidance “FDA and Industry Actions on Premarket Notification (510(k)) Submissions: Effect on FDA Review Clock and Goals” (43).

Regarding nucleic acid-based tests, the Center for Devices and Radiological Health (CDRH) at FDA has extensive experience with clearing and approving this device type. Assay developers are encouraged to use the Agency’s publicly available database that contains 510(k) submissions on this device type (e.g., microbial tests) to determine if an appropriate predicate for their submission can be established (44). This resource also provides the corresponding SE determination Decision Summaries that potential sponsors may review as guidance towards appropriate device specific studies (e.g., analytical and clinical validation). A relevant example of SE determination for an agent detection device is the cleared Joint Biological Agent Identification and Diagnostic System (JBAIDS) Anthrax Detection System (45). Additionally, FDA has published a guidance document, entitled “Highly Multiplexed Microbiological/Medical Countermeasure In Vitro Nucleic Acid-Based Diagnostic Devices—Guidance for Industry and Food and Drug Administration Staff,” that may be of particular relevance to developers of nucleic acid-based agent diagnostic devices (46). This document recommends studies (e.g., analytical) for establishing the performance characteristics of Highly Multiplexed Microbiological Devices (HMMDs). FDA considers these recommended studies to be relevant for premarket notifications [e.g., 510(k) or de novo (*see* proceeding section on De Novo for further information on this regulatory pathway)].

Note that assays involving biothreat agents/MCMs are limited in what is publicly disclosed in decision summaries by nature of the sensitivity of information contained in these device type submissions. As such, sponsors are encouraged to discuss with FDA any submission concerns or specific requirements that may not be informed through decision summary reviews (*see* aforementioned presubmission program).

#### 6.2.3 *De Novo* Classification Process

With the modification to section 513(f)(2) of the FD&C Act through the FDA Safety and Innovation Act (FDASIA), sponsors that believe their device is appropriate for classification into Class I or Class II and determines, based on currently available information, *there is no legally marketed predicate device*, can submit a *De Novo* request without a preceding 510(k) and Not Substantially Equivalent (NSE) decision (i.e., “Direct *De Novo*”). The *De Novo* request must include a description of the device and detailed information and reasons for any recommended classification. If the requester demonstrates that the criteria at section 513(a)(1)(A) or (B) of the FD&C Act are met, the Agency will grant the *De Novo* request, in which case the specific device and device type is classified in Class I or Class II. The granting of the *De Novo* request allows the device to be marketed immediately, creates a classification regulation for devices of this type, and permits the device to serve as a predicate device.

FDA will review De Novo requests for devices that are not within a device type that has been classified under the criteria at section 513(a)(1) of the FD&C Act. This includes devices that do not fall within any existing classification regulation, where the De Novo requester either determines that there is no predicate device or has received an NSE determination on a 510(k) submission. If the device is within a type for which there is an existing classification regulation or one or more approved PMAs, the appropriate mechanism for classification into Class I or II would be reclassification under section 513(e) or section 513(f)(3) of the FD&C Act. Additional information on the De Novo classification process can be found in the Agency’s guidance “De Novo Classification Process (Evaluation of Automatic Class III Designation).”

#### 6.2.4 Premarket approval

A device may be classified in class III and be subject to PMA via several different regulatory vehicles. In accordance with the criteria at section 513(a)(1)(C) of the FD&C Act, FDA may promulgate a regulation classifying, or issue an order reclassifying, a device type into Class III based on the risks posed by the device and the inability of general and special controls to provide reasonable assurance of the safety and effectiveness of the device. All particular devices of this type are considered to be in Class III. Further information on the PMA process can be found on the Agency’s website (47, 48).

## 7 Conclusions

Traditional molecular assay development pipeline involves *in silico* process only in early stages of assay design and relies heavily on wet lab testing for optimization and validation of assay designs. Because of the multiple iterations of wet lab testing needed, this approach is arduous, costly, time consuming, and may result in suboptimal assays.

The modern assay development pipeline proposed here relies heavily on extensive *in silico* approaches up front using a variety of bioinformatic tools and well-curated genome sequence databases to design robust assays and guide wet lab testing. Thus, this process reduces the time and cost involved in developing new molecular assays or improving old assays by minimizing wet lab testing guided by *in silico* results.

We describe best practices for wet lab testing and validation of molecular assays for diagnostics application and monitoring of assay degradation over time due to signature erosion.

We also provide broad guidelines for creating data and documentation for successful submission for regulatory reviews. We have also provided a glossary of terms and definitions commonly used in molecular assay development to aid assay developers.

## 8 Glossary

(**a**) *Accuracy.—*Closeness of agreement between a quantity value obtained by measurement and the true value of the measurand [from VIM 2012 (49)]. This is essentially the rate of the assay giving the correct result. Assays with high accuracy have low occurrence of both false positives and false negatives. The equation for accuracy is given by:

$$\mathrm{Accuracy}=\;\frac{TP+TN}{TP+TN+FP+FN}$$

where TP = true positives, TN = true negatives, FP = false positives, and FN = false negatives.

(**b**) *PCR assay design*.—An *in silico* process to select the assay components such as primers and probe.

(**c**) *Assay development*.—Entire process from target identification/selection, assay design, validation, optimization, matrix testing, preparation of the packages for DoD acceptance, FDA EUA, FDA 510(k), etc.

(**d**) *Assay improvement*.—Any improvement (*in silico* or wet lab testing or both) of existing assays in response to a variety of outcomes from initial testing or field testing. For example, signature erosion, failure of assays in certain conditions or specific matrices, or in multiplex formats.

(**e**) *Assay optimization*.—*In silico* optimization of parameters.

(**f**) *Assay optimization (wet lab)*.—Wet lab testing of the designs from the *in silico* process under different conditions of PCR (concentrations of components, temperature, etc.).

(**g**) *Assay performance*.—Overall assessment of the assay, including sensitivity, specificity, and LOD of an assay in a particular sample matrix.

(**h**) *Assay stewardship (assay performance monitoring)*.—*In silico* validation of assay performance with the availability of new genomic sequences over time.

(**i**) *Assay validation*.—Comprehensive analysis of the test system (i.e., sample to answer process including steps such as extraction, testing, and analysis) to define the assay performance characteristics in the intended matrix. The test system is only validated for use in that specific matrix.

(**j**) *Background panel*.—Panel of organisms found in the typical matrix of the sample (e.g., human genome or human microbiome for human samples, soil microbes for environmental samples, etc.).

(**k**) *Bias*.—Difference between the expectation of the test result or measurement result and the true value [from ISO 3534-2 (50)].

(**l**) *Certified Reference Material (CRM)*.—Reference material accompanied by documentation issued by an authoritative body and providing one or more specified property values with associated uncertainties and traceability, using valid procedures [from VIM 2012 (49)].

(**m**) *Data packages*.—DoD-specific CB56 or FDA-specific EUA or 510(k) body of data that contains all the required information and data.

(**n**) *Exclusivity*.—Nontarget agents, which are potentially cross-reactive, but are not expected to be detected by the method.

(**o**) *Exclusivity panel*.—Panel of near neighbors that are expected to be negative for the assay. Exceptions (i.e., false positives) are expected and need to be tested.

(**p**) *Guideline*.—General rule, principle, or piece of advice; a piece of information that suggests how something should be done—there is some inducement to follow these.

(**q**) *Inclusivity*.—Strains or isolates or variants of the target agent(s) that the method can detect.

(**r**) *Inclusivity panel*.—Panel of strains of the intended PCR assay target organism to include members that represent the organism’s entire genetic diversity. Ideally, the assay is expected to be positive (i.e., sensitive) for all the panel strains. Exceptions (i.e., false negatives) are expected and need to be tested.

(**s**) *Intended use*.—Use for which a product, process, or service is intended according to the specifications, instructions, and information provided by the manufacturer [from ISO 14971 (50)].

(**t**) *Limit of detection (LOD)*.—Lowest concentration or mass of analyte in a test sample that can be distinguished from a true blank sample at a specified probability level.

(**u**) *Limit of quantitation (LOQ)*.—Lowest level of analyte in a test sample that can be reasonably quantified at a specified level of precision.

(**v**) *Matrix*.—Totality of components of a material system except the analyte [from ISO 17511 (50)].

(**w**) *Matrix testing.—*Testing the final assay in specific matrices relevant to the end user; e.g., clinically relevant matrices, such as blood, sputum, etc. or environmentally relevant matrices, such as soil.

(**x**) *Matrix panel*.—Alternate term for background panel (*see* above for definition).

(**y**) *Metrology*.—Science of measurement and its application [from VIM 2012 (49)].

(**z**) *Near neighbors*.—Organisms and/or substances selected to be either closely related or potentially cross-reactive with the organism and/or substance under test. These are targets that are likely to give a false positive for a given assay.

(**aa**) *Probability of detection (POD)*.—Proportion of positive analytical outcomes for a qualitative method for a given matrix at a given agent level or concentration. POD is concentration dependent.

(**bb**) *Recommendation.—*Suggestion or proposal as to the best course of action, especially one put forward by an authoritative body, that is beneficial to follow.

(**cc**) *Reference material (RM)*.—Material that is sufficiently homogeneous and stable with reference to specified properties, which has been established to be fit for its intended use in measurement or in examination of nominal properties [from VIM 2012 (49)]. For purposes of assay development, RMs are templates used in development, testing, validation, and test and evaluation of the assay; e.g., live organisms, inactivated organisms, genomic materials (DNA or RNA), synthetic plasmids, or even synthetic amplicons.

(**dd**) *Robustness*.—Study that tests the capacity of a method to remain unaffected by small but deliberate variations in method parameters and which provides an indication of its reliability during normal usage [from USP 1225 (51)].

(**ee**) *Sample (material)*.—Batch of matrix from which replicate test portions are removed for analysis. The sample (uncontaminated or contaminated) contains agent at one specified level.

(**ff**) *Sensitivity*.—True positive rate, which is the fraction of actual positive samples that the assay returns a positive result. This is also called the “probability of detection.” Assays with high sensitivity have a low occurrence of false negatives. Sensitivity should not be confused with LOD. The equation for sensitivity is given by:

$$\mathrm{Sensitivity}=\frac{TP}{TP+FN}$$

where TP = true positives and FN = false negatives.

(**gg**) *Signature*.—One or more oligonucleotide sequences (i.e., forward primer and/or reverse primer and/or probe) that experimentally detects (e.g., by PCR or probe-based methods) most or all of the desired target organism sequences (i.e., inclusivity panel) and does not detect most or all nontarget sequences (exclusivity panel, near neighbors, and environmental panel). This definition deviates from the traditional definition of a signature as a single oligonucleotide sequence that is “present” in all targets and “absent” in all nontargets. This traditional definition is problematic due to the various definitions of “presence” and “absence” depending on the threshold parameters set for example, (1) edit distance; (2) *BLAST* alignment score; (3) melting temperature or delta G; (4) impact of 3’ terminal primer mismatches.

(**hh**) *Signature erosion*.—Emergence of mutations in the sequences of the signature (primers and probes)that may lead to assay failure. This may happen via the natural course of evolution, especially in viruses, due to genetic drift or shift or deliberate acts.

(**ii**) *Specificity*.—True negative rate is the fraction of tests that are true negative divided by the actual number of negative samples (i.e., true negative plus false positive). Assays with high specificity have a low occurrence of false positives. The equation for specificity is given by:

$$\mathrm{Specificity}=\frac{TN}{TN+FP}$$

where TN = true negatives and FP = false positives.

(**jj**) *Target selection*.—Entails identifying suitable “unique regions” in the genome of interest for assay design. These regions may be longer than the actual PCR amplicon.

(**kk**) *Test and evaluation (T&E)*.—Process by which a system or components are tested and results analyzed to provide performance related information. The results provide information to identify risks, empirical data for validation, and assessment of technical performance, specifications, system maturity, and suitability for intended use.

(**ll**) *Test portion*.—Quantity of subsample or member of a sample set that is taken for analysis by the method.

(**mm**) *Validation*.—Establishment of the performance characteristics of a method and provision of objective evidence that the performance requirements for a specified intended use are fulfilled [ISO 16140-1:2016 (50)].

## Review and approval

The guidelines were reviewed by the AOAC Stakeholder Panel on Agent Detection Assays (SPADA) and approved on October 15, 2019.

## FUNDING

This work was cofunded by DBPAO, JPEO, JPL EB and DoD DUSA T&E.

## Disclaimer

Certain commercial equipment, instruments, or materials are identified in this paper only to specify the experimental procedure adequately. Such identification is not intended to imply recommendation or endorsement by NIST, nor is it intended to imply that the materials or equipment identified are necessarily the best available for the purpose.

The content of this publication does not necessarily reflect the views or policies of the Department of Health and Human Services, nor does the mention of trade names, commercial products, or organizations imply endorsement by the U.S. government. This publication is not an official FDA guidance or policy statement.
